# National trends in drug-related hospitalizations and registered drug-related deaths in Ecuador, 2004–2024: a nationwide serial cross-sectional study

**DOI:** 10.3389/fpubh.2026.1845384

**Published:** 2026-08-03

**Authors:** Esteban Ortiz-Prado, Juan S. Izquierdo-Condoy, Jorge Vasconez-Gonzalez, María Gabriela Davila

**Affiliations:** One Health Research Group, Faculty of Medicine, Universidad de las Américas, Quito, Ecuador

**Keywords:** drug policy, drug possession, Ecuador, interrupted time-series, joinpoint regression, public health, thresholds

## Abstract

**Background:**

Ecuador implemented a threshold-based drug possession policy in 2013 to distinguish personal possession from trafficking-related offenses. This policy was repealed in November 2023. Although threshold-based policies are often discussed within public health and harm-reduction frameworks, their effects cannot be inferred from hospitalization data alone.

**Objective:**

This study describes national patterns of drug-related hospitalizations and registered drug-related deaths in Ecuador using administrative records from 2004 to 2024. We additionally applied quasi-experimental and trend methods (joinpoint regression, interrupted time-series, and individual-level logistic regression) to explore whether patterns changed in association with the 2013 policy.

**Methods:**

We conducted a nationwide serial cross-sectional descriptive analysis of administrative hospital discharge records and registered mortality data from the National Institute of Statistics and Censuses of Ecuador (INEC). Drug-related records were identified using selected ICD-10 categories (F11–F16, F18, F19). Annual hospitalization rates per 100,000 population and registered drug-related deaths were summarized descriptively; Wilson 95% confidence intervals were calculated for proportions. Joinpoint regression characterized trend changes; segmented quasi-Poisson interrupted time-series (ITS) models with a population offset estimated level and slope changes at 2013 (with the November 2023 repeal and the COVID-19 period assessed in sensitivity analyses); and a logistic regression on all hospital discharges (2010–2024) estimated the adjusted odds that a discharge was drug-related before versus after 2013.

**Results:**

Drug-related hospitalization rates increased from 2.479 per 100,000 in 2004 to 7.311 per 100,000 in 2024, with the highest observed rate in 2019 (11.935 per 100,000). A total of 164 registered drug-related deaths were identified during 2004–2024 (151 males, 92.1%; 95% CI 86.9–95.3). F19 remained the most frequent category (107 deaths, 65.2%). In the ITS model, the 2013 implementation was associated with an immediate increase in the hospitalization-rate level [incidence rate ratio (IRR) 2.14; 95% CI 1.22–3.76; *p* = 0.008], without a significant change in slope. Joinpoint analysis identified rising hospitalization rates [annual percentage change (APC) +11.3% through ~2018] and accelerating mortality from ~2014 (APC +26.8%). In the individual-level analysis, discharges after 2013 had higher adjusted odds of being drug-related (adjusted odds ratio 2.64; 95% CI 2.50–2.79; 2.17 after adjustment for the secular trend).

**Conclusion:**

Administrative records show an increasing recorded hospital burden associated with drug-related disorders in Ecuador. Although the quasi-experimental analyses showed a statistically significant change in level coinciding with 2013, these models lack a control group and cannot establish causality; the estimates remain associational and are vulnerable to secular trends, coding changes, and concurrent events, notably COVID-19.

## 1 Introduction

The evolution of drug policy in Ecuador, marked by the implementation of the “Drug Threshold Table” in 2013 and its withdrawal in November 2023, encapsulates the intricate challenges of drug policy reform globally (Table 1). This policy initiative, mirroring strategies in developed countries like Canada, Switzerland, Norway, and the United States, sought to distinguish between personal possession for use and possession for trafficking purposes. The rationale behind such policies lies in the recognition of drug addiction as primarily a public health issue rather than solely a matter of criminal justice, suggesting a paradigm shift in the approach to drug-related offenses (1, 2).

**Table 1:** Ecuador's legal thresholds for narcotic and psychotropic substances (Updated 2015) (12).

Scale (grams)	Narcotic substances							
	Heroin		Cocaine paste		Cocaine hydrochloride		Marijuana	
	Minimum	Maximum	Minimum	Maximum	Minimum	Maximum	Minimum	Maximum
**Minimum**	>0	0.1	>0	2	>0	1	>0	20
**Medium**	0.1	0.2	2	50	1	50	20	300
**High**	0.2	20	50	2.000	50	5.000	300	10.000
**Large**	>20		>2.000		>5.000		>10.000	
Psychotropic substances								
Scale (grams)	Amphetamines		Methylenedioxyphenethylamine (MDA)		Ecstasy (MDMA)			
	Minimum	Maximum	Minimum	Maximum	Minimum		Maximum	
**Minimum**	>0	0.090	>0	0.09	>0		0.090	
**Medium**	>0.09	2.5	>0.09	2.5	>0.09		2.5			
**High**	>2.5	12.5	>2.5	12.5	>2.5		12.5			
**Large**	>12.5		>12.5		>12.5			

Ecuador's adoption of the Drug Threshold Table was framed as a measure to reduce the criminalization of people who use drugs and to align national drug policy with a more health-oriented approach. However, legal reforms of this type require accompanying public health strategies, including prevention, harm reduction, early intervention, treatment access, and continuous monitoring. Without such complementary measures, administrative health records may reveal increasing pressure on hospital services, although these patterns cannot by themselves establish causality or measure the true population burden of substance use disorders (3).

During the decade in which the threshold framework was in place, public and academic debate in Ecuador remained divided regarding its implications. While the policy was intended to support a more humane and rational approach to drug possession, concerns emerged about whether it was implemented without sufficient public health infrastructure, prevention programs, and treatment capacity (4, 5). In this context, changes in hospitalization patterns, mortality records, and the age profile of hospitalized patients can provide useful descriptive information. However, such registry-based indicators should be interpreted cautiously, as they reflect recorded healthcare events rather than population-level substance use incidence or direct policy effects.

The repeal of the threshold policy in November 2023 opened a new chapter in Ecuador's drug policy narrative and created an opportunity to examine the patterns observed during the years in which the policy was in force (6). This development raises broader questions about the extent to which legislative reforms can contribute to public health objectives when they are not accompanied by robust health-system responses, surveillance mechanisms, and accessible services for prevention, treatment, and harm reduction.

International experiences further illustrate the complexity of threshold-based and decriminalization-oriented drug policies. In Portugal, a national law enacted in 2001 decriminalized the use and possession of all drugs, including cocaine and heroin, treating personal use as an administrative rather than criminal offense (7). Portuguese decriminalization has been associated with no major increase in drug use, as well as reductions in problematic consumption, drug-related harms, and pressure on the criminal justice system (8). Nevertheless, subsequent analyses have also highlighted ongoing challenges, including changes in enforcement, institutional capacity, and the need to sustain human-rights-oriented and health-centered responses over time (9).

By examining Ecuador's experience within this broader global and regional context, this study aims to describe national patterns of drug-related hospitalizations and registered drug-related deaths during the threshold-policy period. Rather than estimating causal effects, the analysis provides a registry-based approximation of the hospital and mortality burden captured by national administrative records, highlighting the importance of coupling legal reforms with robust public health initiatives to address substance-related harms.

## 2 Methodology

### 2.1 Study design

This study was designed as a nationwide serial cross-sectional descriptive analysis of drug-related hospital admissions and registered deaths in Ecuador between 2004 and 2024. Data were obtained from the Instituto Nacional de Estadística y Censos (INEC). The hospitalization series was constructed from the complete national discharge database (2010–2024) and combined with previously published rates for 2004–2009; the mortality series covers 2004–2024 in full.

The analysis was not designed to estimate the causal effect of Ecuador's Drug Threshold Table. No internal control group, external control group, or controlled counterfactual comparison was available. Therefore, all temporal comparisons are interpreted as descriptive registry-based patterns rather than causal effects of the threshold policy.

### 2.2 Study setting and population

This study was conducted in Ecuador, a country divided administratively into 24 provinces and 221 cantons and characterized by four main geographic regions: the Coast, the Andean Highlands, the Amazon region, and the Galápagos Islands. The study population included all hospital discharge records and registered deaths coded under the selected ICD-10 substance-related disorder categories between 2004 and 2024.

Records were eligible for inclusion regardless of age, sex, nationality, province, or canton of residence. The analysis was restricted to cases captured in administrative hospital discharge and mortality records and therefore represents registry-based healthcare and mortality events rather than the full population burden of substance use disorders.

### 2.3 Data source

This study used administrative hospital discharge records and registered mortality data obtained from the INEC for the period 2004–2024. Hospital discharge records were used to estimate annual drug-related hospitalization rates, while mortality records were used to describe registered drug-related deaths during the same period. All data were publicly available, anonymized, and aggregated administrative records; therefore, no individual-level identifiers or clinical charts were accessed. Annual hospitalization rates were computed using INEC national population projections as denominators; these denominators reproduce the previously published rates (e.g., 2019 = 11.935 per 100,000). For 2023–2024, post-census INEC projections (2024 revision) were used. Because the early hospital extraction available for 2004–2009 was incomplete, the published 2004–2009 rates were retained, and 2005–2009 values were confirmed against the original year-by-year estimates.

Drug-related records were identified using available three-character ICD-10 categories related to mental and behavioral disorders due to psychoactive substance use. The included categories were F11, disorders due to opioid use; F12, disorders due to cannabinoid use; F13, disorders due to sedative or hypnotic use; F14, disorders due to cocaine use; F15, disorders due to the use of other stimulants, including caffeine; F16, disorders due to hallucinogen use; F18, disorders due to volatile solvent use; and F19, disorders due to multiple drug use and use of other psychoactive substances.

F17, corresponding to disorders due to tobacco use, was excluded because tobacco was not among the substances targeted by Ecuador's threshold-based drug possession policy. The main longitudinal analysis was conducted using three-character ICD-10 groupings because this was the level of diagnostic aggregation consistently available across years and data sources.

Because F15 includes other stimulants, including caffeine, stimulant-related findings should be interpreted with caution. The available administrative data did not allow caffeine-specific diagnoses to be separated from other stimulant-related disorders. However, in the mortality dataset analyzed for this study, F15 did not contribute registered deaths during the study period.

The available data also limited the consistent use of fourth-character ICD-10 subcategories across the full longitudinal series. Therefore, the main analysis could not systematically distinguish acute intoxication, harmful use, dependence syndrome, withdrawal states, psychotic disorders, or unspecified disorders across all years. A complementary four-character ICD-10 tabulation was reviewed separately to provide additional descriptive information on the clinical composition of available substance-related records.

### 2.4 Data analysis

Descriptive outcomes (annual hospitalization rates, registered deaths, age and sex distributions, and ICD-10 categories) were summarized using frequencies, percentages, means, and annual rates. Ninety-five percent confidence intervals for proportions were calculated using the Wilson score method.

Province-level mortality and hospitalization rates were calculated per 100,000 person-years, and 95% confidence intervals for these rates were calculated assuming a Poisson distribution.

We performed three pre-specified analyses. First, joinpoint regression on log-transformed rates identified the location and magnitude of trend changes, reporting the annual percentage change (APC) per segment and the average annual percentage change (AAPC), with a permutation-type F test for the presence of a joinpoint (10). Second, segmented (interrupted time-series) regression estimated changes in level and slope at the a priori intervention point of 2013, modeling annual counts with a Poisson/quasi-Poisson distribution and a log-population offset; overdispersion was addressed with a quasi-Poisson scale, and residual autocorrelation was screened with the Durbin–Watson statistic (11). The November 2023 repeal and the COVID-19 period (2020–2022) were evaluated as additional terms in sensitivity analyses. Third, an individual-level logistic regression on all hospital discharges (2010–2024; *n* = 15,811,994) modeled the odds that a discharge was drug-related (F11–F16, F18, F19 = 1) as a function of the post-2013 period, adjusting for age and sex, with and without a continuous calendar-year term to separate the step change from the underlying trend. Analyses were performed in Python (statsmodels) and SPSS v26.0; two-sided *p* < 0.05 was considered significant.

These models constitute a quasi-experimental, ecological design without a control group. The estimates quantify associations between the timing of the policy and the observed indicators; they do not establish causation and remain susceptible to confounding by secular trends, drug-market changes, diagnostic coding, health-system access, and concurrent events.

### 2.5 Ethical considerations

The CEISH-UDLA reviewed and exempted this study from further ethical evaluation, as per the exemption letter coded 2023-EXC-008. This exemption acknowledged the use of publicly available, anonymized INEC data, aligning with ethical standards for secondary data analysis and obviating the need for Institutional Review Board approval. The study's adherence to ethical research principles was further ensured by the anonymity of the data and compliance with legal norms.

## 3 Results

### 3.1 Descriptive profile of registered drug-related deaths

Between 2004 and 2024, 164 drug-related deaths were registered. Most occurred among males (151 deaths, 92.1%; 95% CI 86.9–95.3), whereas 13 occurred among females (7.9%; 95% CI 4.7–13.1). The most frequent category was F19 (107 deaths, 65.2%; 95% CI 57.7–72.1), followed by F11 (23; 14.0%), F14 (17; 10.4%), F16 (6; 3.7%), F12 and F18 (4 each; 2.4%), and F13 (3; 1.8%). No deaths were registered under F15 (Table 2).

**Table 2:** Descriptive profile of registered drug-related deaths, Ecuador, 2004–2024.

Characteristic	*n*	% (95% CI)
Total registered deaths 2004–2024	164	100
Male	151	92.1 (86.9–95.3)
Female	13	7.9 (4.7–13.1)
F19 Multiple/other psychoactive	107	65.2 (57.7–72.1)
F11 Opioids	23	14.0 (9.5–20.2)
F14 Cocaine	17	10.4 (6.6–16.0)
F16 Hallucinogens	6	3.7 (1.7–7.8)
F12 Cannabinoids	4	2.4 (1.0–6.1)
F18 Volatile solvents	4	2.4 (1.0–6.1)
F13 Sedatives/hypnotics	3	1.8 (0.6–5.2)
F15 Other stimulants	0	0.0 (0.0–2.3)

Wilson 95% confidence intervals; denominator *n* = 164. Values updated for the 2004–2024 period. The complementary four-character ICD-10 profile (*n* = 62) from the original extraction is unchanged.

Updated annual counts include 17 deaths in 2023 and 23 deaths in 2024, the latter being the highest annual count of the series. The 2020–2024 period accounted for 92 of the 164 deaths (56.1%; 95% CI 48.4–63.5). When examined by policy period, 26 deaths occurred in 2004–2012 (15.9%) and 138 in 2013–2024 (84.1%), corresponding to annual averages of 2.9 and 11.5 deaths per year, respectively.

A complementary four-character ICD-10 tabulation (*n* = 62) from the original extraction indicated that dependence syndrome was the most frequent clinical subcategory (31 records, 50.0%), followed by unspecified disorders, harmful use, acute intoxication, and withdrawal states. This complementary profile was not re-extracted for 2023–2024 and is retained as descriptive context.

### 3.2 Drug-related hospitalization rates over time

**Figure 1:**
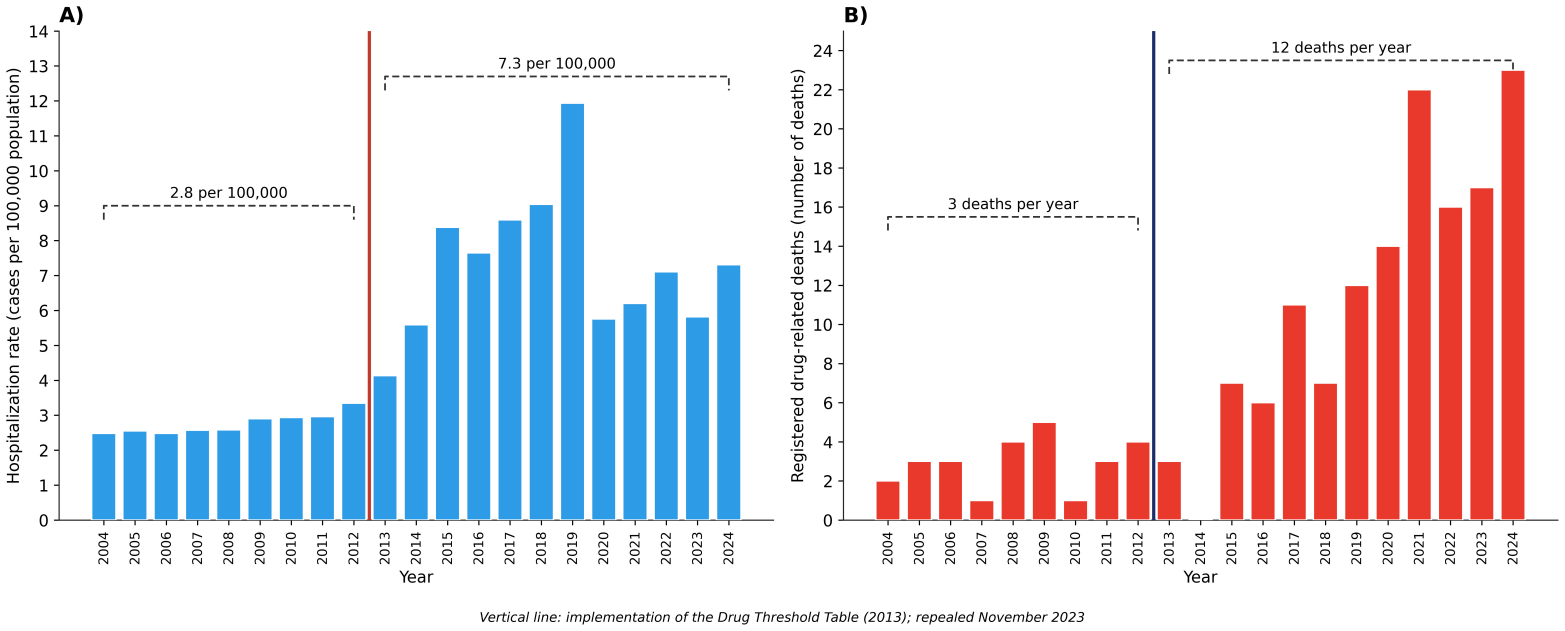
Drug-related hospitalization rates and registered drug-related deaths in Ecuador, 2004–2024. **(A)** Annual hospitalization rates per 100,000 population. **(B)** Annual number of registered drug-related deaths. The vertical line indicates the 2013 implementation of the Drug Threshold Table, repealed in November 2023. The figure is descriptive and should not be interpreted as evidence of a causal policy effect.

National administrative records showed a fluctuating but generally increasing pattern. The drug-related hospitalization rate increased from 2.479 per 100,000 in 2004 to 7.311 per 100,000 in 2024, with the highest observed rate in 2019 (11.935 per 100,000). The average annual rate rose from approximately 2.8 per 100,000 before 2013 to approximately 7.3 per 100,000 during the threshold period (Figure 1A). After the 2019 peak, rates declined during 2020–2021 and remained moderate through 2024 (5.816 in 2023; 7.311 in 2024).

### 3.3 Registered drug-related deaths over time

Registered deaths increased descriptively over the period, from 2 in 2004 to 23 in 2024. The highest annual counts occurred in 2024 (23), 2021 (22), 2023 (17), and 2022 (16) (Figure 1B). These differences should be interpreted as registry-based temporal patterns.

### 3.4 Province-level geographic variation

Province-level analyses showed substantial geographic heterogeneity in registered drug-related hospitalization and mortality patterns. The highest drug-related hospitalization rates were observed in Galápagos, Guayas, Santa Elena, Cañar, Pichincha, Azuay, and Los Ríos, whereas the lowest rates were observed in Carchi, Cotopaxi, Orellana, Sucumbíos, Loja, and Esmeraldas. Registered drug-related mortality rates were highest in Santa Elena, Manabí, Los Ríos, and Guayas. However, province-level mortality findings should be interpreted cautiously because the absolute number of deaths was small in most provinces, resulting in unstable estimates and wide confidence intervals (Table 3).

**Table 3:** Province-level registered drug-related mortality rates, 2004–2024, and drug-related hospitalization rates, 2010–2024, Ecuador.

Province	Deaths, *n*	Mortality person-years	Mortality rate/100,000	95% CI	Discharges, *n*	Discharge person-years	Discharge rate/100,000	95% CI
Azuay	8	16,759,186	0.048	0.021–0.094	1,011	12,580,714	8.04	7.55–8.55
Bolívar	1	4,186,141	0.024	0.001–0.133	88	3,067,456	2.87	2.30–3.53
Cañar	0	5,386,519	0.000	0.000–0.068	342	4,014,630	8.52	7.64–9.47
Carchi	0	3,741,064	0.000	0.000–0.099	46	2,735,443	1.68	1.23–2.24
Cotopaxi	1	9,452,677	0.011	0.000–0.059	135	7,042,255	1.92	1.61–2.27
Chimborazo	2	10,379,320	0.019	0.002–0.070	325	7,646,854	4.25	3.80–4.74
El Oro	4	13,973,521	0.029	0.008–0.073	450	10,329,627	4.36	3.96–4.78
Esmeraldas	3	12,309,945	0.024	0.005–0.071	265	9,243,911	2.87	2.53–3.23
Guayas	66	84,400,914	0.078	0.060–0.099	6,071	63,071,271	9.63	9.38–9.87
Imbabura	2	9,217,854	0.022	0.003–0.078	224	6,860,999	3.26	2.85–3.72
Loja	1	10,282,110	0.010	0.000–0.054	217	7,577,115	2.86	2.50–3.27
Los Ríos	15	17,841,788	0.084	0.047–0.139	868	13,305,118	6.52	6.10–6.97
Manabí	32	30,904,459	0.104	0.071–0.146	774	22,799,870	3.39	3.16–3.64
Morona Santiago	0	3,592,154	0.000	0.000–0.103	168	2,752,599	6.10	5.22–7.10
Napo	0	2,466,120	0.000	0.000–0.150	56	1,884,390	2.97	2.24–3.86
Pastaza	0	2,051,141	0.000	0.000–0.180	73	1,587,052	4.60	3.61–5.78
Pichincha	9	60,764,256	0.015	0.007–0.028	3,815	45,897,004	8.31	8.05–8.58
Tungurahua	1	11,556,467	0.009	0.000–0.048	395	8,557,631	4.62	4.17–5.09
Zamora Chinchipe	0	2,227,616	0.000	0.000–0.166	74	1,692,197	4.37	3.43–5.49
Galápagos	0	602,276	0.000	0.000–0.612	184	463,460	39.70	34.17–45.87
Sucumbíos	1	4,206,006	0.024	0.001–0.132	75	3,235,154	2.32	1.82–2.91
Orellana	1	3,019,156	0.033	0.001–0.185	52	2,316,615	2.24	1.68–2.94
Santo Domingo de los Tsáchilas	4	8,602,409	0.046	0.013–0.119	204	6,521,017	3.13	2.71–3.59
Santa Elena	13	7,374,985	0.176	0.094–0.301	504	5,643,021	8.93	8.17–9.75

Mortality rates were calculated using registered drug-related deaths from 2004–2024. Hospitalization rates were calculated using complete hospital discharge records from 2010–2024. Rates are expressed per 100,000 person-years. Confidence intervals were calculated assuming a Poisson distribution. Province-level mortality estimates should be interpreted cautiously because of the small number of registered deaths in several provinces.

### 3.5 Age distribution of drug-related hospital admissions

**Figure 2:**
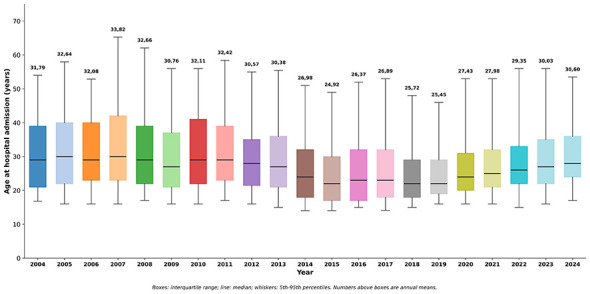
Age distribution of drug-related hospital admissions in Ecuador, 2004–2024. Boxes represent the interquartile range, horizontal lines the medians, and whiskers the observed spread; numbers above each box indicate the annual mean age. Years 2010–2024 were recomputed from the complete national discharge database; 2004–2009 derive from the available early extraction. The figure is descriptive and not evidence of changes in population-level substance use.

Age at hospitalization showed temporal variation. Mean age was generally higher in the early years and lower in several years after 2013, with a modest increase in 2022–2024 (means of 29.3, 30.0, and 30.6 years, respectively) (Figure 2). A decrease in the average age of hospitalization does not necessarily indicate increased drug-use initiation in the general population and may reflect clinical severity, access, or coding factors.

### 3.6 Trend analysis (joinpoint regression)

For hospitalization rates, a single joinpoint was identified near 2018 (*F* = 8.29; *p* = 0.003). The rate rose at +11.3% per year (95% CI 8.4 to 14.1) through ~2018 and declined thereafter (APC −6.4%; 95% CI −16.9 to 5.3; non-significant); the average annual percentage change across 2004–2024 was +7.4% (95% CI 5.0 to 9.8). For registered deaths, a joinpoint near 2014 (*F* = 3.04; *p* = 0.074) separated a flat early segment from an accelerating later segment (APC +26.8%; 95% CI 9.1 to 47.4), with an AAPC of +11.9% (95% CI 6.0 to 18.1).

### 3.7 Interrupted time-series analysis

**Figure 3:**
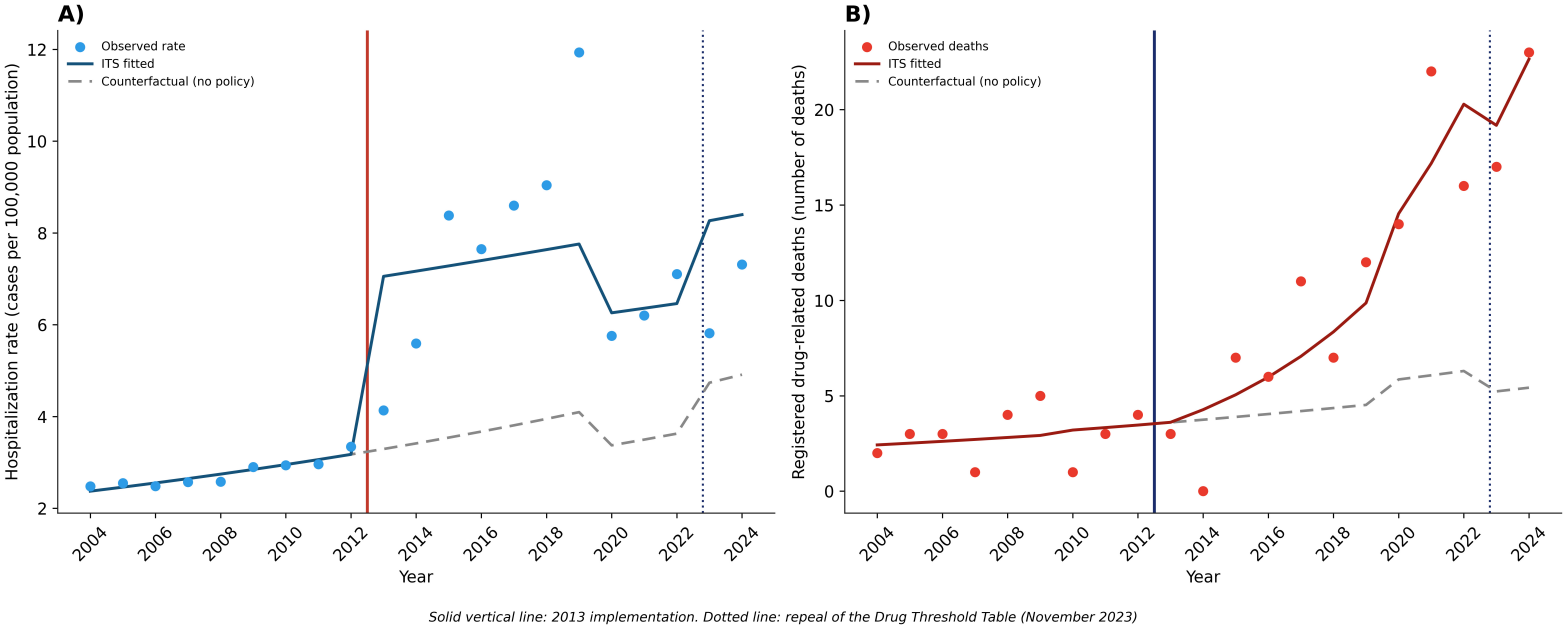
Interrupted time-series analysis of drug-related hospitalization rates **(A)** and registered deaths **(B)** in Ecuador, 2004–2024. Points are observed values; solid lines are model-fitted values from segmented quasi-Poisson regression with a population offset; dashed gray lines are the counterfactual expected in the absence of the 2013 intervention. The solid vertical line marks the 2013 implementation and the dotted line the November 2023 repeal. Estimates are associational and do not establish causality.

In the segmented quasi-Poisson model with a population offset, the 2013 implementation was associated with a significant immediate increase in the hospitalization-rate level (IRR 2.14; 95% CI 1.22–3.76; *p* = 0.008), without a significant change in the annual slope (IRR 0.98; 95% CI 0.88–1.09). The pre-intervention trend was +3.7% per year. For registered deaths, no significant level change was observed at 2013 (IRR 1.00; 95% CI 0.40–2.50), whereas the post-intervention slope was higher than the pre-intervention slope (IRR 1.14 per year; 95% CI 0.97–1.34; *p* = 0.11), consistent with the joinpoint findings (Table 4). The COVID-19 term was not statistically significant in either model. In sensitivity analyses including the November 2023 repeal, the post-repeal terms were unstable and underpowered (only two post-repeal years) and are not interpreted as policy effects. Model fitted values and the corresponding no-intervention counterfactual are shown in Figure 3.

**Table 4:** Summary of inferential models.

Model	Parameter	Estimate (95% CI)	p
ITS hospitalizations	Level change at 2013	IRR 2.14 (1.22–3.76)	0.008
	Slope change after 2013	IRR 0.98 (0.88–1.09)	0.70
ITS deaths	Level change at 2013	IRR 1.00 (0.40–2.50)	0.99
	Slope change after 2013	IRR 1.14 (0.97–1.34)	0.11
Joinpoint hospitalizations	APC to ~2018	+11.3% (8.4–14.1)	< 0.01
Joinpoint deaths	APC from ~2014	+26.8% (9.1–47.4)	< 0.05
Logistic (discharges)	Post-2013, adj. age+sex	aOR 2.64 (2.50–2.79)	< 0.001
	Post-2013, + year trend	aOR 2.17 (2.04–2.32)	< 0.001

IRR, incidence rate ratio; APC, annual percentage change; aOR, adjusted odds ratio. All estimates are associational; the design lacks a control group and does not support causal inference.

### 3.8 Individual-level logistic regression

Among 15,811,994 hospital discharges recorded between 2010 and 2024, 16,426 (0.10%) were drug-related. Adjusting for age and sex, discharges occurring after the 2013 implementation had higher odds of being drug-related [adjusted odds ratio (aOR) 2.64; 95% CI 2.50–2.79]. After additionally adjusting for a continuous calendar-year trend, the post-2013 step remained significant (aOR 2.17; 95% CI 2.04–2.32), alongside a secular annual increase (aOR 1.026 per year; 95% CI 1.021–1.031). Male sex (aOR 8.30; 95% CI 7.99–8.63) and younger age (aOR 0.977 per year of age) were strongly associated with drug-related discharge. Because the comparison contrasts 2010–2012 with 2013–2024 within an upward secular trend, the odds ratios are precise but remain associational and should not be read as causal effects of the policy.

## 4 Discussion

This nationwide registry-based analysis describes temporal patterns in drug-related hospitalization rates and registered drug-related deaths in Ecuador between 2004 and 2024, a period that included the implementation of the Drug Threshold Table in 2013 (1, 12). The recorded hospital burden increased over time, with rates rising from 2.479 per 100,000 in 2004 to 7.311 per 100,000 in 2024 and peaking in 2019. Registered deaths also increased, reaching the highest annual count in 2024 (23 deaths); 56.1% of all deaths occurred during 2020–2024, overlapping the COVID-19 pandemic.

The inferential analyses provide a more formal characterization of these patterns. The interrupted time-series model showed a statistically significant increase in the level of hospitalization rates coinciding with 2013 (IRR 2.14), and the individual-level analysis showed higher adjusted odds that a discharge was drug-related after 2013 (aOR 2.64, attenuating to 2.17 after accounting for the secular trend). Joinpoint analysis, however, located the dominant inflection in hospitalization trend nearer 2018 and the acceleration of mortality nearer 2014, indicating that the observed changes unfolded over several years rather than as an abrupt, isolated shift in 2013. Taken together, these results are compatible with a change in recorded burden during the threshold-policy era but do not isolate the policy from co-occurring influences.

Several factors limit causal interpretation. The design has no control group or counterfactual beyond the model-based extrapolation; overdispersion was substantial for hospitalizations (addressed with a quasi-Poisson scale); and the series comprises only 21 annual points, which constrains power, particularly for the two-change-point and post-repeal analyses. The concentration of deaths in 2020–2024 coincides with the COVID-19 pandemic, which independently affected health-service access, treatment availability, and mental health (13–16). Diagnostic coding, drug-market dynamics, the 2016 earthquake, and the 2019 economic crisis may also have contributed. The logistic comparison contrasts a short pre-period (2010–2012) with a long post-period within an upward trend, which can inflate the apparent step. For these reasons, the estimates are best understood as quasi-experimental associations rather than causal effects.

Most deaths were coded as F19 (multiple/other psychoactive substances), followed by opioid- and cocaine-related disorders, suggesting that polysubstance or poorly specified deaths represent a substantial component of the registered burden. The inclusion of F11 is relevant because heroin was part of Ecuador's legal threshold framework (1, 12). Mortality subgroup estimates remain unstable given the small number of deaths (164 over 21 years), and confidence intervals should be read accordingly.

Evidence from other settings also suggests that the design of threshold policies matters. A study in British Columbia compared three models: one defining personal possession by the absence of evidence of trafficking, one using a cumulative threshold of 15 grams, and one using a cumulative threshold of 2.5 grams. The analysis showed that threshold models can either promote or limit the stated objectives of decriminalization depending on how possession limits are defined and operationalized (17). In Latin America, policy approaches remain heterogeneous: drug possession or use remains criminalized in countries such as El Salvador, Colombia, and Bolivia, although some degree of informal decriminalization may occur, whereas Mexico has decriminalized possession of small amounts of cannabis, cocaine, and other drugs (18).

The Ecuadorian experience should be interpreted within the broader international debate on threshold-based and decriminalization-oriented drug policies. Portugal's experience has been cited as evidence that decriminalization can reduce the burden on the criminal justice system and support a more health-oriented response when accompanied by broader institutional and public health measures (7–9). However, threshold-based policies are not inherently protective or harmful; their effects depend on policy design, implementation, enforcement, and the availability of health and social services (17, 19). Evidence from Latin America also shows heterogeneous public and policy attitudes toward drug reform, suggesting that national context is central to how these policies are interpreted and implemented (18).

Evidence from other settings also suggests that changes in drug policy may be accompanied by changes in healthcare utilization patterns. For example, studies of cannabis policy liberalization in the United States have reported changes in emergency department presentations related to cannabis or other drug-related episodes (20, 21). These findings do not imply that similar mechanisms operated in Ecuador, but they reinforce the need to monitor health-service utilization alongside legal reforms.

As contextual information, the geographic distribution of registered inpatient drug rehabilitation centers is shown in the Supplementary Figure 1. The map illustrates the location and institutional type of these facilities, including Ministry of Health, municipal, and private fee-for-service centers. Although the distribution appears uneven and visually concentrated in selected urban areas, this information should be interpreted cautiously. The present study did not evaluate facility capacity, occupancy, affordability, accessibility, quality of care, or treatment outcomes. Therefore, Supplementary Figure 1 should be understood as contextual health-system information and not as direct evidence explaining the observed hospitalization or mortality patterns.

Overall, these findings support the need for integrated drug policy strategies that combine legal clarity with prevention, harm reduction, early intervention, accessible treatment, and improved surveillance. Such an approach is consistent with international recommendations emphasizing that substance use disorders require coordinated responses across health systems, social services, and justice sectors (2, 19, 22). More rigorous evaluations using linked health, mortality, emergency care, toxicology, criminal justice, and population-level data are needed to determine the health effects of threshold-based drug policies in Ecuador and comparable settings.

### 4.1 Limitations

This study has several limitations related to the use of administrative registry data. Hospital discharge records capture only cases that reached inpatient care and therefore cannot be interpreted as the true population incidence or prevalence of substance use disorders. These data exclude individuals managed in outpatient care, emergency departments without admission, community settings, private services not fully captured in administrative records, or those who did not seek care.

Although we incorporated joinpoint regression, segmented interrupted time-series models, and individual-level logistic regression to formally characterize temporal patterns, causal interpretation remains limited. The study did not include an internal control group, an external control group, or a robust counterfactual comparison. Therefore, the estimates should be interpreted as associational registry-based patterns rather than causal effects of the Drug Threshold Table.

Several unmeasured contextual factors may have influenced hospitalization and mortality patterns during the study period. These include changes in healthcare access, hospital admission practices, diagnostic coding, drug markets, drug toxicity, socioeconomic conditions, the 2016 earthquake, the 2019 economic crisis, the COVID-19 pandemic, and broader security or public health dynamics. In particular, the concentration of registered deaths during 2020–2024 coincided with the COVID-19 pandemic and may reflect pandemic-related disruptions in healthcare, treatment access, mental health, and drug supply rather than policy effects.

The mortality analysis was limited by the small number of registered drug-related deaths. Only 164 deaths were identified over the full study period, and some subgroup counts by sex, province, and ICD-10 category were very small. Therefore, proportions, rates, and confidence intervals for these subgroups should be interpreted only as descriptive summaries of the registry data and not as stable population-level mortality estimates or subgroup-specific risk estimates.

Diagnostic specificity was also limited by the use of three-character ICD-10 categories. The main longitudinal analysis could not distinguish acute intoxication, harmful use, dependence syndrome, withdrawal states, psychotic disorders, or unspecified disorders across all years. Although a complementary four-character ICD-10 tabulation was reviewed, these data were not consistently available with annual disaggregation across all included categories and therefore could not serve as the basis for the longitudinal analysis.

An additional classification limitation is that F15 includes other stimulants, including caffeine, and the available administrative data did not allow caffeine-specific diagnoses to be separated from other stimulant-related disorders. Although F15 did not contribute registered deaths in the mortality dataset, this remains a potential source of misclassification for stimulant-related hospitalization records. Likewise, category F19 includes cases of multiple substance use, as well as situations in which the substance responsible for the clinical presentation cannot be identified with certainty or in which patterns of use are indiscriminate. Consequently, this group may be influenced by differences in clinical documentation and coding practices.

It is also important to note that the database reports cases according to ICD-10 classification and does not provide information on toxicological confirmation, hospital care processes, emergency department encounters without admission, or outpatient management. Therefore, it was not possible to verify the substance involved in each case or to assess the full spectrum of substance-related healthcare utilization. Furthermore, the database does not include indicators of clinical severity, such as intensive care unit admission, length of hospital stay, complications, or treatment response. As with other administrative databases, underreporting or incomplete case classification may have occurred, potentially leading to an underestimation of the hospital burden.

Finally, although we added province-level hospitalization and mortality rates to describe geographic heterogeneity, these analyses remain ecological and descriptive. Provincial mortality estimates are particularly unstable because of the small number of registered deaths in several provinces. Differences across provinces may reflect variation in population structure, healthcare access, hospital admission practices, coding behavior, service availability, and care-seeking patterns rather than true differences in substance-related morbidity or mortality. Future studies should incorporate more granular geographic, clinical, toxicological, emergency care, outpatient, criminal justice, and population-based data to better characterize regional differences and potential mechanisms underlying the observed patterns.

## 5 Conclusion and policy implications

This nationwide serial cross-sectional analysis describes drug-related hospitalization rates and registered drug-related deaths in Ecuador during the period in which a threshold-based drug possession policy was implemented and later repealed. The findings suggest an increasing recorded hospital burden, with registered deaths concentrated mainly among males and among records coded as multiple drug use, opioid-related disorders, and cocaine-related disorders. Complementary four-character ICD-10 data indicated that dependence syndrome was the most frequent clinical subcategory among available disaggregated records.

These findings are descriptive and do not establish a causal relationship between the threshold policy and drug-related hospitalization, mortality, or population-level substance use. The small number of registered deaths further limits the stability of mortality subgroup estimates, even when confidence intervals are provided. Future evaluations should integrate hospital, emergency, outpatient, toxicological, criminal justice, and population-based data, ideally using quasi-experimental approaches with appropriate comparison groups.

The observed registry patterns support aligning legal reforms with prevention, harm reduction, early intervention, accessible treatment, and strengthened surveillance, consistent with international recommendations for embedding drug policy reform within broader public health and health-system responses.

## Data Availability

The original contributions presented in the study are included in the article/Supplementary material, further inquiries can be directed to the corresponding author.
