# Supplementary material for: National trends in drug-related hospitalizations and registered drug-related deaths in Ecuador, 2004–2024: a nationwide serial cross-sectional study
Source: Front Public Health. 2026 Aug 3;14:1845384. doi: 10.3389/fpubh.2026.1845384 (PMC13478205; doi:10.3389/fpubh.2026.1845384)
Supplement: Supplementary file 1 [file Image_1.pdf]

Supplementary Material

Supplementary Material

1.1 Supplementary Figures

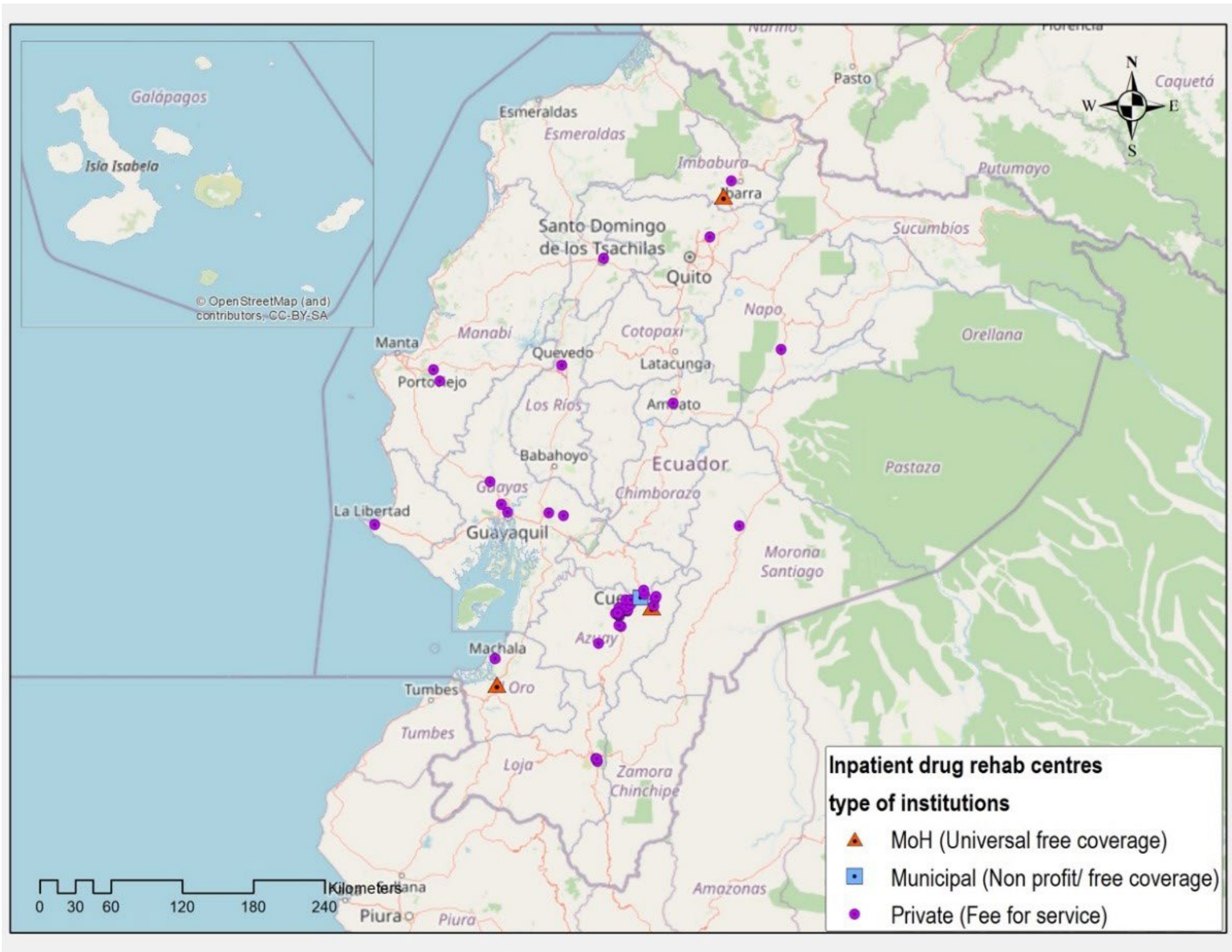

Supplementary Figure 1. Geographic distribution of registered inpatient drug rehabilitation centers in Ecuador. The map shows the location and institutional type of registered inpatient drug rehabilitation centers, including Ministry of Health, municipal, and private fee-for-service facilities. This figure provides contextual health-system information and should not be interpreted as a measure of treatment capacity, accessibility, quality of care, or causal explanation for hospitalization patterns.
